# Available Biomarkers for Personalized Prognostication in Early and Very Early Systemic Sclerosis: A Narrative Review of the Current Literature

**DOI:** 10.3390/jpm16070355

**Published:** 2026-06-30

**Authors:** Isabel Dirven, Marre W. Kamminga, Lise M. Verhoef, Rogier M. Thurlings, Ruben L. Smeets, Arjan van Caam, Madelon C. Vonk

**Affiliations:** 1 Department of Rheumatology, Radboud University Medical Center, 6525 GA Nijmegen, The Netherlands; isabel.dirven@radboudumc.nl (I.D.); marre.kamminga@radboudumc.nl (M.W.K.); or l.verhoef@maartenskliniek.nl (L.M.V.); rogier.thurlings@radboudumc.nl (R.M.T.); arjan.vancaam@radboudumc.nl (A.v.C.); 2 Department of Rheumatology, Sint Maartenskliniek, 6574 NA Nijmegen, The Netherlands; 3 Department of Laboratory Medicine—Medical Immunology, Radboud University Medical Center, 6525 GA Nijmegen, The Netherlands; ruben.smeets@radboudumc.nl; 4 Radboudumc Laboratory for Diagnostics, Department of Laboratory Medicine, Radboud University Medical Center, 6525 GA Nijmegen, The Netherlands

**Keywords:** early systemic sclerosis, very early systemic sclerosis, VEDOSS, progression, biomarkers, interstitial lung disease, skin involvement, mortality, Raynaud’s phenomenon

## Abstract

**Background:** Systemic sclerosis (SSc) is a heterogeneous autoimmune disease characterized by inflammation, vasculopathy, and fibrosis. It is associated with the highest mortality among rheumatic diseases. Very early SSc may represent a critical phase with risk of developing progressive disease. Although timely treatment may be effective in patients with progressive disease, it carries risks of adverse events, underscoring the need for early identification of individuals at risk. Biomarkers for progression in the early stage offer opportunities for timely intervention and improved long-term outcomes. Therefore, validating biomarkers that predict progression is an important research priority. In this narrative literature review, we summarize and evaluate blood circulating biomarkers associated with different progression endpoints in (very) early SSc. **Methods:** The literature search was conducted using PubMed. Eligible studies assessed biomarkers in very early SSc or early SSc cohorts with longitudinal follow-up and progression-related outcomes. **Results**: The identified studies investigated biomarkers associated with interstitial lung disease (ILD), skin progression, overall disease progression, and mortality. Anti-topoisomerase I was associated with ILD development. A high interferon score was linked to reduced lung function and mortality. KL-6 was associated with progression in early SSc-ILD. PRO-C3 and PRO-C6 showed the strongest associations with skin involvement. Finally, IgG anti-centromere antibody was associated with organ involvement and progression to definite SSc. CXCL10 and TNFRII were linked to progression and significant survival differences in the discovery and replication cohorts. However, effect sizes were often modest, and findings were inconsistent across cohorts. Substantial heterogeneity in study design, populations, endpoints, and biomarker assessment methods limited comparability. Moreover, most biomarkers demonstrated associations at the group level but lacked sufficient discriminatory power for individual risk prediction. Only a minority of studies included validation cohorts, and replication of findings was limited. **Conclusions**: Multiple biomarkers show promising associations with progression in very early and early SSc, but a single biomarker is unlikely to reliably predict disease progression.

## 1. Introduction

Systemic sclerosis (SSc) is a rare systemic autoimmune disease characterized by inflammation, vasculopathy, and fibrosis, with a prevalence of 200 people per million worldwide [1]. The prognosis of SSc patients has improved over the past 30 years, with five-year survival rates up to 80% [2]. However, in patients with progressive disease, a three-year survival rate is as low as 50% [3,4]. The exact pathogenesis is unknown to date; however, it has been hypothesized that an unknown trigger leads to endothelial injury in small vessels, activating both innate and adaptive immune responses [5]. Immune-derived profibrotic mediators subsequently drive fibroblast–myofibroblast differentiation, resulting in excessive extracellular matrix deposition and progressive fibrosis of the skin and internal organs, including the heart, lungs, and gastrointestinal tract (GI). These collectively contribute to the high mortality associated with SSc [6].

Very early SSc is increasingly recognized as a critical phase in the disease course, as timely treatment may improve outcomes. This phase is associated with a high risk of developing progression, including skin thickening, musculoskeletal involvement, interstitial lung disease (ILD), renal disease, and increased mortality [7]. Clinically, this phase often presents with new-onset Raynaud’s phenomenon (RP) and/or puffy fingers, typically in the absence of skin fibrosis or internal organ involvement. The concept of “early SSc” was first defined by Leroy and Medsger in 2001, based on the presence of RP, SSc-specific autoantibodies (anti-centromere (ACA), anti-Th/To, anti-topoisomerase I (ATA), or anti-RNA polymerase III (ARA), and/or a characteristic nailfold capillaroscopy (NCM) pattern [8]. In 2011, the European Scleroderma Trials and Research group (EUSTAR) and the European Alliance of Associations for Rheumatology (EULAR) refined these criteria into the Very Early Diagnosis of Systemic Sclerosis (VEDOSS) by adding puffy fingers to the criteria [9,10].

Close monitoring of patients during this early stage possibly offers a valuable opportunity to improve long-term outcomes by enabling timely therapeutic intervention. Although available therapies primarily aim to reduce inflammation, mitigate fibrosis, and alleviate symptoms, they are often associated with substantial adverse effects, such as increased risk of infections. This underscores the importance of identifying patients who are most likely to benefit from early or intensive treatment approaches [11]. Early targeting of vascular or cutaneous manifestations may help prevent the development of serious complications such as ILD or pulmonary arterial hypertension (PAH), which are among the leading causes of SSc-related mortality [12]. A study by D. Haverkort et al. [13], showed that 43% up to 73% of patients fulfilling the amended VEDOSS criteria had disease progression over a 3-year follow-up [13]. Accordingly, an important priority for future research is the identification and diagnostic validation of early biomarkers capable of predicting progressive disease. Incorporating such biomarkers into treatment algorithms could facilitate personalized and preventive strategies [12].

Given the growing interest in identifying biomarkers associated with progression in very early SSc, multiple studies have investigated markers linked to various progression outcomes. In this narrative review, we summarize and evaluate blood circulating biomarkers associated with different progression endpoints in VEDOSS and early SSc, with the aim of evaluating their predictive value for disease progression.

## 2. Methods

**Figure 1:**
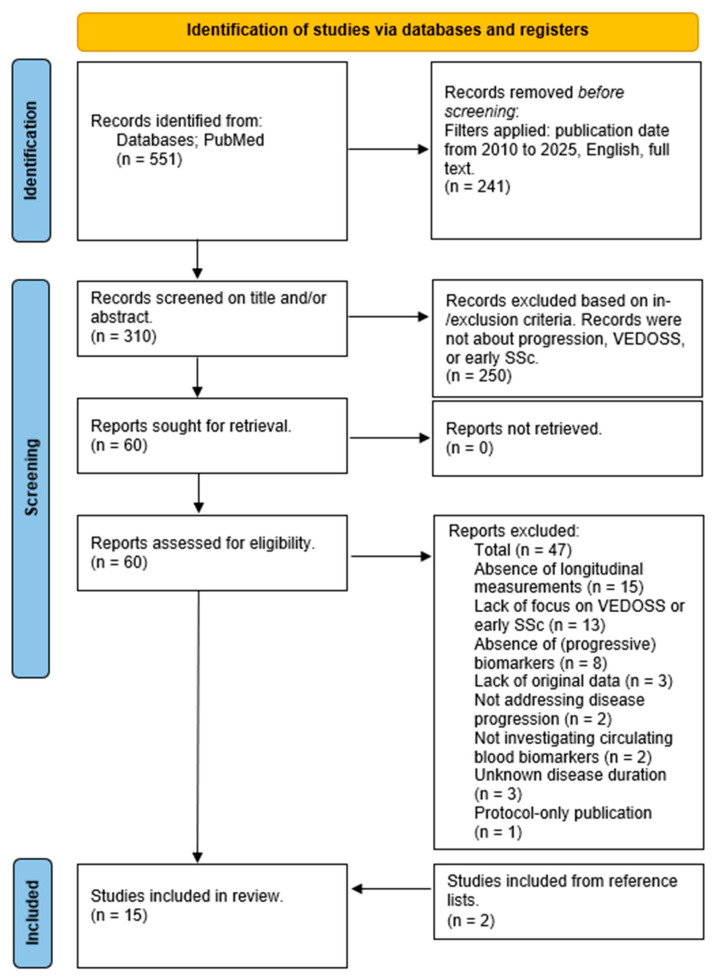
Flowchart literature search.

The primary objective of this narrative review was to provide an overview and evaluation of prognostic circulating blood biomarkers for disease progression in VEDOSS and early SSc. Given the heterogeneity of study designs, methodologies, and outcome measures across the available literature, a non-systematic approach was adopted. Consequently, the review protocol was not registered in PROSPERO or other dedicated databases. Nevertheless, a PRISMA flow diagram is provided (Figure 1). In addition to database searches, reference lists of relevant publications were also manually screened to identify further eligible studies. 

The PubMed database was searched for publications from 2010 onwards to 2025, as the VEDOSS criteria were first introduced in 2010 [14]. The search strategy included Medical Subject Headings (MeSH) terms referring to ((Systemic sclerosis) AND (Early)) AND (Biomarker) (Appendix A). Articles were screened by title and/or abstract, followed by full-text assessment by two independent reviewers; disagreements were resolved through consensus. In addition to database searches, the reference lists of relevant publications were manually screened to identify additional eligible studies. Studies were included if they addressed blood circulating biomarkers, VEDOSS, or early SSc, involved multiple measurement time points, were available in full text, and were written in English. Studies were excluded if disease duration was >5 years since the first non-RP symptom, or if they were reviews, cross-sectional studies, or meta-analyses.

## 3. Results

The search strategy initially identified 551 potentially eligible articles. After applying filters for publication date (2010–2025), English language, and full-text availability, 310 articles remained. A total of 241 articles were excluded during the initial filtering stage. The remaining 310 articles were screened based on title and abstract, focusing on studies addressing disease progression, early SSc, or VEDOSS. This resulted in 60 articles being selected for full-text review.

Following full-text assessment, 13 articles met the inclusion criteria. A total of 47 studies were excluded for the following reasons: lack of focus on VEDOSS or early SSc (*n* = 13), unknown disease duration (*n* = 3), not investigating circulating blood biomarkers (*n* = 2), absence of (progressive) biomarkers (*n* = 8), not addressing disease progression (*n* = 2), absence of longitudinal measurements (*n* = 15), lack of original data (*n* = 3), and protocol-only publication (*n* = 1).

In addition to the database search, two additional studies were identified through manual screening of reference lists (snowballing). These studies were not retrieved in the initial search, as they did not explicitly mention early SSc or VEDOSS; however, the reported disease duration was consistent with early SSc (Figure 1).

The included studies were characterized based on the type of progression outcomes, how these outcomes were assessed, and the biomarkers evaluated. All studies employed a cohort design, and the included studies were conducted across 11 countries worldwide. Per the study, population size varied, ranging from 21 to 625 participants. Some studies focused on VEDOSS, others on early SSc, and some on a combination of both. Mean or median disease duration since the first non-RP symptom was reported in almost all studies involving early SSc patients. Follow-up duration was consistently reported across studies, although expressed differently, either as mean or median values (Table 1).

## 4. Discussion

### 4.1. Biomarkers Associated with Interstitial Lung Disease

ILD affects up to 80% of patients with SSc and represents a leading cause of SSc-related mortality [12]. Approximately 25–30% of patients with ILD develop a progressive phenotype that may ultimately result in respiratory failure and death. Disease progression of SSc-ILD is typically defined by a ≥10% predicted decline in forced vital capacity (%FVCpred), or a ≥5% predicted decline accompanied by a decrease in diffusion capacity for carbon monoxide (DLCO) of ≥15% predicted, or radiological progression on high-resolution computed tomography (HRCT) [30,31,32].

Several biomarkers have been proposed as potential predictors of ILD development or progression in early SSc. Among SSc-specific autoantibodies, anti-topoisomerase I (ATA) is most consistently associated with ILD. The study by Assassi et al. [29], demonstrated that ATA positivity correlated with a more rapid decline in FVC over the first three years of follow-up, suggesting a more aggressive ILD progression. However, this association was not maintained in long-term analyses without time restriction, potentially reflecting survival bias, as patients with rapidly progressive, fatal ILD may have been underrepresented in extended follow-up cohorts. Notably, ATA was assessed as a dichotomous variable, without consideration of antibody titers. Supporting this, Mulalin et al. [33], reported that higher ATA levels were associated with earlier onset of ILD in Thai patients with SSc. Furthermore, across multiple cohorts, ATA positivity has consistently been identified as a risk factor for ILD development [34]. Although Assassi et al. [29], used a longitudinal model to assess predictors of FVC decline in a relatively large cohort (GENISOS, *n* = 266), there could be bias towards ATA effects. ATA is associated with diffuse cutaneous SSc (dcSSc), which may be relevant given that 60% of the cohort has dcSSc, and ATA is known to be more prevalent in patients with dcSSc [29]. However, Hasegawa et al. [27] observed a reduced %FVC at the third year in ATA-positive patients versus negative patients on the group level in a Japanese cohort (*n* = 93) [27]. This strengthened the role of ATA as a biomarker.

KL-6, a glycoprotein produced by type II pneumocytes, has also emerged as a promising biomarker for SSc-ILD [19,23]. Both Salazar et al. [19] and Kuwana et al. [23] have identified KL-6 as a predictor of progression in early SSc-ILD, including progression to end-stage lung disease. Despite these associations at the group level, substantial inter-individual variability has been observed: some patients with low KL-6 levels develop ILD, while others with elevated levels remain stable. This variability limits its predictive accuracy for individual patients [19,23]. Collectively, current evidence suggests that KL-6 performs well as a diagnostic biomarker for ILD in SSc. However, it remains uncertain whether its sensitivity and specificity are equally robust when applied as a prognostic biomarker for ILD progression in early SSc-ILD.

In addition, inflammatory markers have been investigated as potential biomarkers for ILD progression in early SSc. In a retrospective study, Hinchcliff et al. [16] showed that several interferon (IFN)-inducible chemokines (CCL8, CCL19, CXCL9, CXCL10) were markedly elevated in dcSSc compared with controls, forming a composite interferon score that stratified patients into IFN-high and IFN-low groups. The elevated IFN score was associated with impaired lung function, higher disability, and increased five-year mortality. The IFN score could potentially serve as a biomarker. However, as this was the first study evaluating the IFN score, further research is necessary to evaluate between biological and analytical variability of the Luminex assay. The study results could be compared with the established mRNA-based IFN signatures, and the IFN score could be assessed longitudinally to determine its behavior over time in the blood. Finally, it may be of interest to assess this score in a limited cutaneous comparator group [16].

CCL2 was not included in the IFN score of Hinchcliff et al. [16] due to the lack of significant differences between dcSSc patients and healthy controls in their study. In contrast, Wu et al. [22] reported that elevated CCL2 levels were associated with a more rapid long-term decline in FVC, whereas IL-10 correlated with a slower decline in the GENISOS cohort, which consists of 266 early SSc patients, including 156 patients with dcSSc. However, these findings were not replicated in an independent validation cohort, highlighting the challenges of reproducibility. Strengths include a large sample size and consistent laboratory methods, with adjustment for known risk factors. A key limitation is that not all patients underwent CT scanning; ILD progression was diagnosed through FVC decline alone [22]. Additional biomarkers, including CHI3L1, in a study by Cossu et al. [21], have been associated with fibrotic lung involvement and reduced DLCO. While cell adhesion molecules were evaluated by Hasegawa et al. [24], they have not demonstrated consistent predictive value as a biomarker for ILD progression [24].

Finally, Ayla et al. [15] examined type VI collagen degradation and formation markers (C6M, PRO-C6), which correlated with ILD severity. C6M was significantly higher in ILD patients, and both C6M and PRO-C6 showed weak negative correlations with FVC% at baseline (R^2^ < 0.4). Despite statistical significance, these effects limit their practical diagnostic value. The collagen biomarkers (PRO-C3, PRO-C6, C3M, C6M) are biological variables, which makes it difficult to measure reliably. They did not predict lung function decline, limiting the current value of ILD prognostication [15].

### 4.2. Biomarkers Associated with Skin Involvement

Skin involvement is one of the hallmark features of SSc. The extent and trajectory of skin involvement serve as important indicators of disease severity and progression. Clinically meaningful progression is commonly defined as a ≥5-point or ≥25% increase in the Modified Rodnan Skin Score (mRSS) from baseline [35].

Various biomarkers have been evaluated as potential predictors of skin disease progression in early SSc. As observed in ILD, autoantibodies play a central role, although their predictive power varies considerably. In the study by Herrick et al. [20], a predictive model for mRSS progression, including ARA, achieved an accuracy of 71.0%. This study was strengthened by a large (ESOS, *n* = 293), well-defined cohort with standardized three-monthly assessments. However, the model relied predominantly on clinical variables, incorporating only a single biomarker (ARA), and did not evaluate independent biomarker effects or adjust for potential confounders [20].

In contrast, Hasegawa et al. [27] reported no prognostic value for autoantibodies such as ATA and ACA with respect to skin progression in a Japanese cohort (*n* = 93) of patients with dcSSc or ILD. Instead, a higher baseline erythrocyte sedimentation rate (ESR), a marker of systemic inflammation, showed a tendency to correlate with mRSS at three-year follow-up, suggesting that inflammatory activity may be more relevant for predicting skin involvement in Japanese SSc patients [27].

Beyond autoantibodies, periostin, a protein involved in tissue repair and fibrotic processes, was investigated by De Luca et al. [17] as a potential prognostic marker in very early SSc. Although periostin levels were elevated in patients compared with controls, no association with progression of skin involvement was observed. Interpretation of these findings is limited by the small sample size (*n* = 15), and further studies in larger cohorts are required to clarify their prognostic relevance [17].

Similarly, collagen markers have shown associations with skin fibrosis. The collagen biomarkers PRO-C3 and PRO-C6 were significantly elevated in patients with dcSSc and demonstrated weak but significant positive correlations with mRSS, indicating their association with the extent of skin involvement according to the study by Ayla et al. [15]. Yet, as with ILD, these correlations did not exceed R^2^ < 0.4, suggesting limited ability to distinguish skin severity between individual patients. Longitudinally, only PRO-C3 predicted mRSS change, but in the opposite direction of cross-sectional associations, likely reflecting early disease peaks in rapidly progressive phenotypes. No other collagen biomarker predicted skin progression, and changes in collagen markers did not correlate with mRSS trajectories over six months, limiting their value for dynamic monitoring. Although their associations with inflammatory markers support biological relevance, further validation is needed before these markers can be clinically applied [15].

### 4.3. Biomarkers Associated with Prognosis and Mortality

Beyond ILD and skin involvement, several biomarkers have been investigated for their ability to predict overall disease progression, transition to definite SSc, and mortality. Among these, autoantibodies appear to have prognostic relevance in very early SSc. Van Leeuwen et al. [18] demonstrated that baseline IgG ACA levels were significantly higher in patients who progressed to definite SSc compared with non-progressors. Elevated levels of multiple ACA isotypes (IgG, IgM, IgA) were associated with organ involvement and disease progression. However, substantial overlap between the populations of progressors and non-progressors limits individual predictive accuracy. The cutoff values of 84% of progressors and 49% of non-progressors were correctly classified. IgG ACA levels therefore show association with disease progression, but predictive performance remains insufficient for clinical use. Additionally, the small sample size and absence of a validation cohort limit robustness [18].

Markers of systemic inflammation have also demonstrated prognostic potential. Liu et al. [25] reported that elevated baseline CRP levels predicted long-term ILD progression, increased mortality, and a more rapid decline in FVC. Notably, CRP levels were particularly elevated in ARA-positive patients not receiving immunosuppressive therapy, suggesting a link between autoantibody profile and inflammatory activation [25].

In addition, inflammatory cytokines have been investigated. Cossu et al. [21] identified CXCL10 and TNFRII as predictors of more rapid progression to definite SSc, with significant survival curves in both discovery and replication cohorts, although cutoff definitions were unclear [21]. Hasegawa et al. [24] demonstrated, in addition to previous research, that ICAM-1 shows an association with ILD, while P-selectin showed potential as a predictor of physical dysfunction, although evidence remains preliminary [24].

Finally, Valentini et al. [26] demonstrated that avascular areas on NCM, elevated PIIINP (a fibroblast activation marker), and increased soluble IL-2Rα (T-cell activation) were independently associated with progression to definite SSc in early disease. However, their ability to predict internal organ involvement was not supported, and the early SSc cohort was small, with limited numbers of true non-progressors [26].

### 4.4. Strengths and Limitations

The findings of this narrative review should be interpreted in light of several limitations. First, publication bias may have influenced the available evidence, as studies reporting negative biomarker findings are less likely to be published. In addition, multiple studies have been conducted within the same cohorts or have evaluated numerous biomarkers simultaneously within a single cohort, increasing the risk of false-positive findings due to multiple testing.

Some studies evaluated biomarkers in both a validation and a replication cohort, such as the study by Cossu et al. [21], which strengthens confidence when consistent results are observed across cohorts. However, in several studies, the predictive performance was not clear, which is important to assess the clinical value of these biomarkers. Overall, although these studies show statistical significance or associations at the population level, such as with KL-6, these findings do not consistently demonstrate meaningful variation at the individual level.

Furthermore, the generalizability of the findings may be limited by the geographical and ethnic composition of the study populations. A substantial proportion of the included studies were conducted in Japanese cohorts. Ethnic differences in the clinical phenotype of SSc have been described in a study by Al-Sheik et al. [36]. They evaluated ethnic variations in the Toronto Scleroderma Program cohort in Canada, where patients self-reported their ethnicity. Ethnicity was categorized as European-descent white, Afro-Caribbean, Hispanic, Arab, East Asian (China, Japan, Korea, Philippines, and Thailand), South Asian (Bangladesh, Nepal, India, Pakistan, and Sri Lanka), First Nations, or Persian. This study indicated that East Asian patients less frequently present with features such as calcinosis cutis, esophageal dysmotility, sclerodactyly, and telangiectasia, while diffuse cutaneous disease may be more common compared with patients of European descent. Despite these phenotypic differences, short-term survival appears to be comparable across ethnic groups, although East Asian patients may have a longer median survival time. Additionally, East Asian patients have been reported to exhibit a lower frequency of calcinosis cutis, while disease duration is similar to that observed in white populations. These differences in disease expression and outcomes suggest that biomarker performance, including their predictive value for ILD development or progression, may vary between populations. Therefore, findings from Japanese cohorts should be interpreted with caution and, where possible, validated in more diverse SSc populations.

Finally, the cohorts included in the reviewed studies are highly heterogeneous, limiting the feasibility of direct comparisons. Considerable variation is observed in effect sizes, study populations, and the biomarkers investigated, which should be taken into account when interpreting the reported associations.

## 5. Conclusions and Future Perspectives

Although several studies suggest potential predictive value of biomarkers for ILD, skin involvement, and mortality, current evidence remains limited, reinforcing the need to prioritize multivariable prediction models over isolated predictors. Given the marked heterogeneity of the disease, a single biomarker is unlikely to reliably predict disease progression.

At present, there remains a substantial unmet need for personalized prognostication to enable treatment strategies tailored to an individual patient’s risk of progression. Recent research has shown that early treatment may improve outcomes [37]. Therefore, initiating appropriate treatment in patients with a short disease duration but an adverse prognosis could help prevent progressive disease. However, as immunomodulatory treatments may be associated with adverse events, more personalized treatment strategies are required. To date, this has not been achievable using a single biomarker; therefore, a combinatorial biomarker approach is warranted. This approach should be evaluated in a sufficiently large cohort of patients with early-stage disease, such as in the Hit Hard and Early trial [38], and subsequently externally validated.

Future research should therefore focus on large, multicenter, and standardized cohorts with uniform follow-up to validate combinations of candidate biomarkers into predictive models, including their external validation in independent populations. Integrating molecular, serological, and clinical markers in translational studies may further enhance personalized risk stratification, early intervention, and targeted therapeutic approaches. Ultimately, robustly validated biomarker panels could enable earlier identification of high-risk patients, guide monitoring intensity, and support more individualized treatment strategies.

## Figures and Tables

**Table 1:** Overview of the study characteristics of the included articles.

Author, Year	Progression Outcome	Assessment Outcome	Assessed Biomarkers	Cohort	Country	Population Size	Study Population	Disease Duration Since First Non-RP	Follow-Up Time	References
Ayla et al., 2025	ILD, skin involvement	mRSS, FVC%	PRO-C3, PRO-C6, C3M and C6M	Prospective	US	*n* = 222	Early SSc	2.7 ± 1.4 years (mean ± SD)	23.2 ± 14.0 months (mean ± SD)	[15]
Hinchcliff et al., 2025	ILD, skin involvement, quality of life, and mortality.	mRSS, FVC%, DLCO%, and HAQ-DI	CCL2, CCL8, CCL19, CXCL9, CXCL10 and CXCL11	Retrospective	US (PRESS) and UK	Total *n* = 182 PRESS *n* = 110 Healthy controls = 32	Early SSc	1.1 (0.7–1.6) years (median; range).	Median: 34 months	[16]
De Luca et al., 2024	Skin involvement	mRSS	Periostin	Prospective	Italy	Total patients *n* = 50 VEDOSS *n* = 15 Healthy controls *n* = 30	VEDOSS	38.2 ± 45.1 months (mean ± SD) in total patients	12 months	[17]
Van Leeuwen et al., 2021	Skin involvement, digital ulcers, ILD, PAH, PH, myocardial involvement, SRC, and GI involvement.	mRSS, FVC%, HRCT imaging, modified Medsger severity scale, RHC, clinical data.	ACA isotope expression.	Prospective	The Netherlands, Norway, Switzerland, Belgium, and France	Total patients *n* = 625 VEDOSS *n* = 138	VEDOSS	NA	Progressor group: median of 5 years (range 3–7) Non-progressor group: median of 2 years (1–5)	[18]
Salazar et al., 2018	ILD	FVC% and DLCO%	KL-6 and CCL-18	Prospective	US (GENISOS cohort)	GENISOS *n* = 82 Healthy controls *n* = 40	Early SSc	2.4 ± 1.5 years (mean ± SD)	12–18 months	[19]
Herrick et al., 2018	Skin involvement	mRSS	mRSS, ATA, ARA, and ACA.	Prospective	EU	ESOS *n* = 293	Early SSc	12.0 (7.0–21.0) months (mean ± SD)	12–24 months	[20]
Cossu et al., 2017	ILD, PAH, digital ulcers, skin involvement, telangiectasias, puffy fingers, and mortality.	FVC%, DLCO%, NVC, RHC.	IL-18, G-CSF, CCL2, CCL4, CCL5, CCL11, CCL19, CXCL9, CXCL10, CXCL11, CXCL13, cathepsin L, cathepsin S, TNFRII, galectin 3, and CHI3L1	Retrospective	Italy	Total patients *n* = 36 EaSSC *n* = 21 Healthy controls *n* = 11 And Total patients *n* = 181 EaSSc *n* = 47 Healthy controls *n* = 43	Early SSc	NA	Maximum follow-up time of 59.6 months	[21]
Wu et al., 2017	ILD	FVC%	IL 1-β, IL-5, IL-6, IL-8, IL-10, IL-12, IL-13, TNF-α, CCL2, I-TAC, and IP-10	Prospective	US (GENISOS cohort) and Canada (CSRG cohort)	GENISOS *n* = 266 CSRG *n* = 171	Early SSc	2.5 years (US) and 2.2 (Canada) years (mean).	Mean GENISOS cohort: 4.36 years (up to 13.1 years). Mean CSRG cohort: 5.72 years (up to 9.71 years).	[22]
Kuwana et al., 2016	ILD	FVC%, DLCO%.	mRSS, ATA, ARA, ACA, KL-6.		Japan	*n* = 50	Early SSc	14.2 ± 7.2 months (mean ± SD)	173.5 ± 64.7 (28–204) months (mean ± SD; range).	[23]
Hasegawa et al., 2014	ILD, skin involvement, PAH, cardiac involvement, and SRC.	mRSS, HAQ-DI, ESR, %VC.	ICAM-1, E-selectin, L-selectin, and P-selectin.	Prospective	Japan	*n* = 92 Healthy controls *n* = 24	Early SSc	19 (1–60) months (median; range).	4 years	[24]
Liu et al., 2013	ILD, muscle involvement, and skin involvement.	FVC%, DLCO%, mRSS, CK.	CRP	Prospective	US	GENISOS *n* = 266 Healthy controls *n* = 97	Early SSc	2.5 ± 1.6 years (mean ± SD)	4.36 (up to 13.1) years (mean).	[25]
Valentini et al., 2012	Telangiectasias, digital ulcers, skin involvement, ILD, GI involvement, and cardiac involvement.	NCM, mRSS, ECG, chest and esophageal X-ray, and lung function tests.	NCM, PIIINP, sIL-2Rα, E-selectin, and ICTP.	Prospective	Italy	*n* = 39	Early SSc	3 (0.5–24) years (median; range).	3 (1–8) years (median; range).	[26]
Hasegawa et al., 2012	ILD and skin involvement.	%VC and mRSS	ATA, ACA, and ESR.	Prospective	Japan	*n* = 93	Early SSc	20 (1–35) months (median; range).	3 years	[27]
Domsic et al., 2010	SRC, ILD, GI, cardiac involvement, and mortality.	Hypertension and/or elevated serum creatinine, pericarditis, myocarditis/cardiomyopathy, conduction abnormalities, FVC%, systolic pressure, oesophageal dysmotility, strictures, hypomotility, malabsorption, colonic sacculations, and mRSS.	ATA, ARA, anti-PM-Scl, anti-Ku, anti-U1RNP, anti-U3RNP, anti-U11/U12RNP, anti-Th/To, and STPR	Prospective	US	*n* = 826	Early SSc	Rapid = 0.5 (0.4–0.6) years (median; range). Intermediate = 0.8 (0.6–1.0) years (median; range). Slow = 1.1 (0.9–1.6) years (median; range).	2 years	[28]
Assassi et al., 2010	ILD	FVC%	ATA, ACA, and ARA.	Prospective	US	GENISOS *n* = 266	Early SSc	2.53 ± 1.63 years (mean ± SD).	3 years	[29]

ACA: Anti-Centromere Antibody, Anti-PM-Scl: Anti-Polymyositis/Scleroderma, ARA: Anti-RNA polymerase III, ATA: Anti-topoisomerase I antibody, Anti-U1RNP: Anti-U1 Ribonucleoprotein, C3M/C6M: Collagen type III/VI M, CCL: CC-Chemokine Ligand, CHI3L1: Chitinase-3-like protein 1, CRP: C-Reactive Protein, CSRG: Canadian Scleroderma Research Group, CXCL: C-X-C motif Chemokine Ligand, DLCO: Diffusing Capacity of the Lungs for Carbon Monoxide, ESOS: European Scleroderma Observational Study, ESR: Erythrocyte Sedimentation Rate, FVC: Forced Vital Capacity, G-CSF: Granulocyte Colony-Stimulating Factor, GENISOS: Genetics versus Environment in Scleroderma Outcomes Study, GI: Gastrointestinal, HAQ-DI: Health Assessment Questionnaire—Disability Index, HRCT: High-Resolution Computed Tomography, I-TAC: Interferon–inducible T Cell Alpha Chemoattractant, ICAM-1: Intercellular Adhesion Molecule-1, ICTP: Carboxy-terminal telopeptide of type I collagen, IL: Interleukin, IP-10: Interferon gamma-induced protein 10, ILD: Interstitial Lung Disease, KL-6: Krebs von den Lungen-6, mRSS: Modified Rodnan Skin Score, NCM: Nailfold Capillary Microscopy, PAH: Pulmonary Arterial Hypertension, PH: Pulmonary Hypertension, PIIINP: N Procollagen III N-terminal Propeptide, Pro-C3/Pro-C6: N-terminal Propeptide of type III/VI collagen, RHC: Right Heart Catheterization, SD: Standard Deviation, sIL-2R: Soluble Interleukin-2 Receptor, SRC: Scleroderma Renal Crisis, SSc: Systemic Sclerosis, STPR: Serum Total Protein obtained by refractometer, TNF-α: Tumor Necrosis Factor α, TNFRII: Tumor Necrosis Factor Receptor II, UK: United Kingdom, US: United States, VC: Vital Capacity, VEDOSS: Very Early Diagnosis of Systemic Sclerosis.

## Data Availability

No new data were created or analyzed in this study. Data sharing is not applicable to this article.

## Abbreviations

The following abbreviations are used in this manuscript:

ACA	Anti-Centromere Antibody
ANA	Antinuclear antibodies
Anti-PM-Scl	Anti-Polymyositis/Scleroderma
ARA	Anti-RNA polymerase III
ATA	Anti-topoisomerase I antibody
Anti-U1RNP	Anti-U1 Ribonucleoprotein
C3M/C6M	Collagen type III/VI M
CCL	CC-Chemokine Ligand
CHI3L1	Chitinase-3-like protein 1
CRP	C-Reactive Protein
CSRG	Canadian Scleroderma Research Group
CXCL	C-X-C motif Chemokine Ligand
dcSSC	Diffuse cutaneous systemic sclerosis
DLCO	Diffusing Capacity of the Lungs for Carbon Monoxide
ESOS	European Scleroderma Observational Study
ESR	Erythrocyte Sedimentation Rate
EULAR	European League Against Rheumatism
EUSTAR	European Scleroderma Trials and Research
FVC	Forced Vital Capacity
G-CSF	Granulocyte Colony-Stimulating Factor
GENISOS	Genetics versus Environment in Scleroderma Outcomes Study
GI	Gastrointestinal
HAQ-DI	Health Assessment Questionnaire—Disability Index
HRCT	High-Resolution Computed Tomography
I-TAC	Interferon–inducible T Cell Alpha Chemoattractant
ICAM-1	Intercellular Adhesion Molecule-1
ICTP	Carboxy-terminal telopeptide of type I collagen
IFN	Interferon
IL1	Interleukin
ILD	Interstitial Lung Disease
IP-10	Interferon gamma-induced protein 10
JBI	Joanna Briggs Institute
KL-6	Krebs von den Lungen-6
MeSH	Medical Subject Headings
mRSS	Modified Rodnan Skin Score
NCM	Nailfold Capillary Microscopy
PAH	Pulmonary Arterial Hypertension
PH	Pulmonary Hypertension
PIIINP	N-Procollagen III N-terminal Propeptide
Pro-C3/Pro-C6	N-terminal Propeptide of type III/VI collagen
RP	Raynaud’s phenomenon
RHC	Right Heart Catheterization
SD	Standard Deviation
sIL-2R	Soluble Interleukin-2 Receptor
SRC	Scleroderma Renal Crisis
SSc	Systemic Sclerosis
STPR	Serum Total Protein obtained by refractometer
TNF-α	Tumor Necrosis Factor α
TNFRII	Tumor Necrosis Factor Receptor II
UK	United Kingdom
US	United States
VC	Vital Capacity
VEDOSS	Very Early Diagnosis of Systemic Sclerosis

## Appendix A

The complete PubMed search string: ((Systemic sclerosis) AND (Early)) AND (biomarker) AND ((fft[Filter]) AND (english[Filter]) AND (2010:2025[pdat])) AND (english[Filter]) AND (2010:2025[pdat]) NOT (Review[Publication Type])).
